# Development of a prognostic model based on immunogenic cell death/ferroptosis-related genes and the study of TREX1 effects on prostate cancer cells

**DOI:** 10.3389/fimmu.2025.1708437

**Published:** 2025-11-19

**Authors:** Yaoan Wen, Shuyuan Zhan, Jianhui Chen, Jiangbin Yang, Dandong Chen, Song Zheng, Shaoxing Zhu

**Affiliations:** 1 Department of Urology, Fujian Medical University Union Hospital, Fuzhou, Fujian, China; 2 Department of Urology, Fujian Provincial Geriatric Hospital, Fuzhou, Fujian, China

**Keywords:** immunogenic cell death, ferroptosis, prostate cancer, prognostic model, TREX1, tumor immune microenvironment

## Abstract

**Background:**

Understanding the interplay between immunogenic cell death (ICD), ferroptosis, and prostate cancer (PCa) is critical for elucidating the underlying mechanisms of PCa pathogenesis. This study aimed to establish a prognostic model for PCa based on ICD- and ferroptosis-related genes (IFRGs) and to evaluate its potential clinical applicability.

**Methods:**

RNA sequencing data and clinical information of PCa patients were obtained from The Cancer Genome Atlas (TCGA-PRAD) database. Candidate IFRGs were identified through Pearson correlation and differential expression analyses. A prognostic model was constructed using univariate Cox regression, least absolute shrinkage and selection operator (LASSO) regression, and Kaplan–Meier survival analyses, and subsequently validated in an external cohort (GSE70769). In addition, siRNA-mediated knockdown of the key gene TREX1 was performed in PC-3 cells, and EdU and Transwell assays were conducted to assess its effects on tumor cell proliferation, migration, and invasion.

**Results:**

A three-gene IFRG-based prognostic model was developed, which effectively stratified PCa patients into high- and low-risk groups with significantly different survival outcomes. Multivariate Cox regression analysis confirmed the model as an independent prognostic factor. Functional experiments further demonstrated that TREX1 serves as a critical risk gene, and its knockdown markedly suppressed the proliferative, migratory, and invasive capacities of PCa cells.

**Conclusion:**

The three-gene IFRG-based prognostic model may serve as a promising prognostic biomarker for PCa, providing predictive value and novel insights into the complex interactions between IFRGs and PCa progression. Moreover, TREX1 was identified as a potential therapeutic target, offering new perspectives for prognostic assessment and the development of immunotherapy strategies in PCa.

## 1 Introduction

Prostate cancer ranks among the most prevalent malignancies affecting men globally. In the United States, projections indicate that approximately 299,000 new cases of prostate cancer will be diagnosed in 2024, representing roughly 29% of all newly identified cancers in the male population (1). The conventional treatment regimen for prostate cancer generally includes surgery and endocrine therapy combined with chemotherapy or radiotherapy (2, 3). Nonetheless, a significant number of patients ultimately advance to castration-resistant prostate cancer (CRPC), a phase marked by an insensitivity to standard therapeutic approaches (4, 5). Unfortunately, diagnostic and prognostic instruments, including prostate-specific antigen (PSA), exhibit significant limitations in accurately predicting outcomes for high-risk patients and may produce false positive results attributable to benign conditions. Consequently, there is a pressing necessity for the development of more dependable prognostic biomarkers and therapeutic targets for prostate cancer.

Immunogenic cell death (ICD) represents a regulated mechanism of cell death that promotes the activation of both innate and adaptive immune responses. This process is characterized by a complex interaction of signaling pathways between dying tumor cells and elements of the immune system (6). During ICD, dying cancer cells release a series of damage-associated molecular patterns (DAMPs), such as calreticulin (CALR), high mobility group box 1 (HMGB1), and ATP (7). These molecules serve as “danger signals” which activate dendritic cells and other antigen-presenting cells, consequently enhancing both innate and adaptive immune responses (8, 9). As a result, ICD facilitates efficient immune surveillance, inhibits tumor advancement, and improves the effectiveness of immunotherapeutic approaches.

Ferroptosis is a form of regulated cell death characterized by its dependence on iron and the mediation of lipid peroxidation, distinguishing it from traditional apoptosis (10). Ferroptosis is defined by the buildup of lipid hydroperoxides resulting from impairments in antioxidant mechanisms, notably those associated with glutathione peroxidase 4 (GPX4) (11). Excessive lipid peroxidation results in irreversible damage to the plasma membrane, ultimately leading to cellular death. Recent studies suggest that ferroptosis may affect the tumor immune microenvironment by releasing damage-associated molecular patterns (DAMPs) and other immunogenic factors from dying cancer cells, thereby stimulating immune responses (11, 12). This enhances anti-tumor immune responses and represents a promising therapeutic strategy when utilized in conjunction with immunotherapies.

Despite their mechanistic differences, ferroptosis and ICD exhibit a significant interrelationship. Ferroptosis facilitates ICD by DAMPs and lipid peroxides. Conversely, immune responses elicited by ICD, including the secretion of interferon-γ by CD8^+^ T cells, can further enhance ferroptosis in tumor cells (12). Existing literature indicates that ferroptosis and ICD are crucial factors in the pathogenesis, therapeutic response, and immune microenvironment associated with prostate cancer (13, 14). Although gene signatures associated with ICD and ferroptosis have been examined separately in the context of prostate cancer, the synergistic impact of these two processes on prognosis and therapeutic outcomes in prostate cancer has not been thoroughly investigated.

In this study, we conducted a comprehensive analysis to identify Immunogenic Cell Death/Ferroptosis-Related Genes (IFRGs) that are associated with prostate cancer, and subsequently developed a prognostic model based on these genes. Additionally, we investigated the expression levels of the model key gene TREX1 in prostate cancer tissues and assessed its functional implications in the proliferation, migration, and invasion of prostate cancer cells. Overall, this study establishes a novel prognostic signature for prostate cancer and elucidates the potential role of ICD/ferroptosis-related pathways, particularly the TREX1-mediated DNA sensing mechanism, in the progression of prostate cancer. This research provides valuable insights for prognostic evaluation and the development of personalized treatment strategies in the context of prostate cancer.

## 2 Materials and methods

### 2.1 Experimental materials

In this study, we conducted an analysis of prostate cancer data utilizing two distinct datasets: TCGA-PRAD and GSE70769. The TCGA-PRAD dataset encompasses information from 549 patients, while the GSE70769 dataset consists of data from 94 patients. Gene and clinical data for the TCGA-PRAD dataset were retrieved from the TCGA database (https://portal.gdc.cancer.gov/). The clinical data includes variables such as age, Gleason score, pathological T stage, N stage, M stage, disease progression status, and duration of disease progression. Additionally, gene expression data along with corresponding clinical information were sourced from the GSE70769 dataset through the GEO database (https://www.ncbi.nlm.nih.gov/geo/).

### 2.2 Data acquisition and preprocessing

The raw data were analyzed utilizing the “LIMMA” package in R software to normalize the gene expression files, adjusting them to fragments per kilobase of transcript per million mapped reads (FPKM). The FPKM data format from The Cancer Genome Atlas Prostate Adenocarcinoma (TCGA-PRAD) dataset was subsequently converted to transcripts per million (TPM).

To maintain the integrity of the analysis, 124 prostate cancer samples with incomplete clinical data were excluded from further clinical-related analyses. We sourced clinical data from the Gene Expression Omnibus (GEO) database (GSE70769), which included 94 prostate cancer samples. Following quality control and normalization procedures, the TCGA-PRAD cohort was designated as the training set, while the GSE70769 cohort served as the validation set for subsequent analyses.

### 2.3 Identification of IFRGs

Through a comprehensive literature review, we identified a total of 44 genes associated with ICD, which were sourced from various published studies (14, 15). The ICD-related genes include: CALR, CASP1, CD4, CD8A, CD8B, CXCR3, EIF2AK3, HMGB1, HSP90AA1, IFNA1, IFNB1, IFNG, IFNGR1, IL10, IL17A, IL17RA, IL1B, IL1R1, IL6, LY96, MYD88, NLRP3, P2RX7, PIK3CA, PRF1, TLR4, TNF, PANX1, ANXA1, FPR1, PDIA3, LRP1, STING1, ENTPD1, NT5E, FOXP3, BCL2, CASP8, CFLAR, PPP1R15A, VTCN1, STC1, CD47, TREX1. Additionally, we retrieved a dataset of 259 genes associated with ferroptosis from the FerrDb V2 database (16) (**Supplementary Table**).

To identify IFRGs in prostate cancer, we conducted a Pearson correlation analysis between the gene sets associated with ICD and ferroptosis, utilizing expression data from TCGA-PRAD. Genes demonstrating significant correlations (|R| > 0.4, P < 0.05) were first identified. Subsequently, differential expression analysis between tumor and normal tissue samples was conducted employing volcano plot methodology with more rigorous thresholds (|fold-change| > 1.3, P < 0.001, FDR<0.05). Genes satisfying both the correlation and differential expression criteria were collectively classified as “IFRGs.” The expression differences of these IFRGs between tumor and normal tissues were then illustrated using boxplot visualizations.

### 2.4 Mutation and copy-number analysis of IFRGs

We conducted an analysis of the mutation status of the IFRGs within the TCGA-PRAD cohort. The somatic mutation data were processed utilizing the “maftools” R package (17) to extract mutation information pertinent to each IFRG. We conducted an analysis of mutation frequency across the samples and created waterfall plots to illustrate the various types and frequencies of mutations observed. To assess copy-number variations (CNVs), we utilized Perl scripts on the TCGA copy-number data to identify gains and losses associated with each IFRG. The frequencies of CNVs (gains versus losses) were visualized, and the chromosomal locations of the IFRGs were plotted accordingly. Furthermore, we developed a protein–protein interaction (PPI) network for the IFRGs using the STRING database to investigate potential functional relationships among these genes. Additionally, we compared the expression of these IFRGs between normal and tumor tissues using limma. The prognostic impact of each gene was evaluated with Kaplan–Meier analysis by stratifying patients into high vs. low expression groups.

### 2.5 Unsupervised clustering and survival analysis

Utilizing the expression profiles of the IFRGs, we conducted consensus clustering of the TCGA-PRAD samples employing the “ConsensusClusterPlus” R package (18) in order to delineate molecular subtypes. The consensus cumulative distribution function (CDF) and delta area plots were employed to ascertain the optimal number of clusters. Following these analyses, the samples were categorized into two distinct clusters, designated as C1 and C2. A Kaplan–Meier analysis was conducted to compare Progression-Free Survival (PFS) rates between these subtypes.

### 2.6 Analysis of the immune microenvironment

To investigate the association between IFRG subtypes and the tumor immune microenvironment, we employed the CIBERSORT algorithm to quantify the relative proportions of various immune cell types present in each tumor sample, and further validated the results using the EPIC and MCP-counter methods. We created bar plots to illustrate the immune cell composition across the samples and generated heatmaps to depict the correlations among the immune cell fractions. We conducted a comparative analysis of immune cell infiltration between high- and low-IFRG subtypes utilizing violin plots, which revealed significant disparities, particularly in the populations of CD8+ T cells, M2 macrophages, and regulatory T cells. Additionally, we analyzed the expression levels of HLA-related genes and immune checkpoint genes across the subtypes using boxplots. We also calculated ESTIMATE scores, which encompass stromal score, immune score, and tumor purity, for each sample through the application of the “ESTIMATE” algorithm (19). Subsequently, we compared these metrics between the different subtypes, assessing variations in tumor purity and the immune/stromal components.

### 2.7 Differential expression and functional enrichment

We conducted a differential expression analysis between the two IFRG-based subtypes utilizing the “limma” package, applying the criteria of |log2 fold change| > 1 and P < 0.05 (20). A total of 1,032 differentially expressed genes (DEGs) were identified in this study. These DEGs were subsequently visualized using a heatmap and a volcano plot. To further analyze the DEGs, Gene Ontology (GO) and KEGG pathway enrichment analyses were performed to ascertain the overrepresented biological processes, cellular components, molecular functions, and pathways, utilizing the clusterProfiler R package (21). In the context of GO, we assessed enrichments across the categories of Biological Process (BP), Cellular Component (CC), and Molecular Function (MF). For KEGG analysis, we identified significantly enriched signaling pathways. Additionally, Gene Set Enrichment Analysis (GSEA) was conducted to compare high-IFRG and low-IFRG groups, aiming to identify hallmark pathways that were enriched in each group.

### 2.8 Construction of the IFRG-based prognostic model

Utilizing the TCGA training dataset, we conducted univariate Cox proportional hazards regression analyses on each of the 18 IFRGs to identify genes that exhibited a significant association with PFS (P < 0.05). This analysis led to the selection of three genes: TREX1, NOX4, and RRM2. Subsequently, we employed LASSO-Cox regression with the glmnet package, which automatically standardizes the input data prior to model fitting, to construct a multivariate prognostic model. The optimal lambda value was established through cross-validation, culminating in the formulation of a final model. The risk score for each patient was computed as a weighted sum of the gene expression levels, represented mathematically as:


$$\sum \left(\text{expression of gene}\times \text{coef}\right)$$

Patients were categorized into high-risk and low-risk groups based on the median risk score. To assess PFS differences between these risk groups, we performed Kaplan–Meier survival analysis within both the TCGA cohort and an independent GEO validation cohort. The predictive accuracy of the model was further evaluated using time-dependent receiver operating characteristic (ROC) curves and the concordance index.

### 2.9 Validation and nomogram construction

To assess the validity of the prognostic model, we visualized the distribution of risk scores alongside the corresponding survival status of patients, and created heatmaps illustrating the expression levels of the three risk-associated genes across different risk groups. Both univariate and multivariate Cox regression analyses were conducted to determine the independence of the risk score in relation to clinical covariates, including Gleason score, tumor stage, and nodal status. A prognostic nomogram was developed utilizing the “rms” package to estimate the probabilities of survival at 1-year, 3-year, and 5-year intervals, based on the risk score and various clinicopathological factors. The predictive accuracy of the nomogram was further evaluated through calibration curves and additional receiver operating characteristic (ROC) analyses.

### 2.10 Immune cell correlation and drug sensitivity analyses

We examined the association between the risk score and the infiltration of immune cells. Spearman correlation analyses were employed to assess the relationship between the risk score and the relative abundances of various immune cell types across the samples. Statistically significant correlations were visualized using scatter plots. Furthermore, drug sensitivity analysis was conducted utilizing the pRRophetic R package (22), which predicts the half-maximal inhibitory concentration (IC50) of chemotherapeutic agents for each sample by integrating gene expression profiles with data from the Genomics of Drug Sensitivity in Cancer (GDSC) database. To address batch effects across datasets, the ComBat algorithm was applied, and ridge regression models were used to estimate drug response.

### 2.11 Somatic mutation and tumor mutation burden

We conducted a comparative analysis of somatic mutation landscapes between high-risk and low-risk cohorts by creating distinct waterfall plots. The most frequently mutated genes were identified. The tumor mutation burden (TMB) was calculated for each individual patient. We then compared TMB across the different risk groups using boxplots and performed Kaplan–Meier survival analyses stratified by TMB (high versus low) and risk group. Survival curves were generated for four subgroups (TMB-high/high-risk, TMB-high/low-risk, TMB-low/high-risk, TMB-low/low-risk) to evaluate the interactions between TMB and risk score in relation to prognosis. We conducted an additional analysis to examine the relationship between TMB and both microsatellite instability (MSI) and programmed death-ligand 1 (PD-L1) expression. The MSI data were obtained from the cBioPortal for Cancer Genomics (https://www.cbioportal.org/). MSI scores were compared between cohorts characterized by high and low TMB to investigate whether elevated mutational loads correspond with increased genomic instability. Furthermore, PD-L1 expression levels were assessed across these TMB-defined groups to explore potential associations between tumor mutational burden and immune checkpoint activation. Differences between groups were statistically evaluated using the Wilcoxon rank-sum test.

### 2.12 TREX1-associated immune microenvironment analysis

Utilizing bioinformatic approaches, TREX1 was identified as the gene with the most significant risk coefficient. Consequently, we conducted an in-depth examination of its expression profiles, immune-related associations, and functional roles. To elucidate the potential involvement of TREX1 in regulating the tumor microenvironment, correlation analyses, immune infiltration assessments, and gene set enrichment analyses were performed.

### 2.13 Immunohistochemistry

To further assess its clinical significance and biological function in prostate cancer, we conducted immunohistochemical staining alongside subsequent functional assays. A commercially obtained tissue microarray (TMA) containing 60 primary prostate cancer samples and 57 adjacent normal prostate tissues was utilized (HProA120Su02, Shanghai Xinchao Biotechnology). The TMA encompassed both prostate cancer tissues and matched adjacent non-tumor tissues, supplemented with comprehensive clinical parameters including patient age, pathological stage, Gleason score, and metastatic status.

The TMA underwent dewaxing and rehydration following standard protocols, which included baking at 63°C for one hour and sequential incubation in xylene, followed by 100%, 90%, 80%, and 70% ethanol. Antigen retrieval was achieved using citrate buffer (pH 6.0) via microwave or water bath methods. Endogenous peroxidase activity was inhibited with 3% H2O2 in methanol for a duration of 10 minutes. Subsequently, the slides were blocked with 5% BSA in PBS for 30 minutes at room temperature. The primary antibody targeting TREX1 (rabbit anti-TREX1 [EPR14985], ab185228, Abcam) was applied at a dilution of 1:200 and incubated overnight at 4°C. The following day, sections were washed with PBS and incubated with HRP-conjugated goat anti-rabbit secondary antibody for one hour at room temperature. After washing, staining was visualized using DAB substrate, and the slides were counterstained with hematoxylin, dehydrated, and coverslipped using mounting medium. TREX1 immunoreactivity was evaluated by two independent pathologists, with cytoplasmic staining scored on a scale of 0 to 3 for intensity (0, negative; 1, weakly positive; 2, intermediately positive; and 3, strongly positive) and 0 to 3 for the percentage of positive cells (0, no staining; 1: <25% cells; 2: 25%–75% cells; and 3: >75% cells), resulting in a combined score ranging from 0 to 9. Samples were classified as exhibiting low expression (0–3) or high expression (4–9). Statistical analysis was performed to compare TREX1 expression levels between cancerous and adjacent tissues using the Chi-square test.

### 2.14 Cell culture and functional assays

The human prostate cancer cell line PC-3 (ATCC CRL-1435) was maintained in RPMI-1640 medium supplemented with 10% fetal bovine serum (FBS) (Gibco) and 1% penicillin-streptomycin, under conditions of 37°C and 5% CO2. For experimental procedures, the cells were plated in suitable culture dishes and subsequently transfected as outlined in the following sections.

### 2.15 siRNA transfection

TREX1-targeting small interfering RNA (siRNA) was synthesized, comprising a sense strand (5′-CCAAGACCATCTGCTGTCA-3′) and an antisense strand (5′-TGACAGCAGATGGTCTTGG-3′). Additionally, a validated non-targeting control siRNA was produced, with a sense strand (5′-UUCUCCGAACGUGUCACGUTT-3′) and an antisense strand (5′-ACGUGACACGUUCGGAGAATT-3′). For the transfection process, PC-3 cells, which were at 60–70% confluence, were treated with 50 nM of the respective siRNA using Lipofectamine 3000 (Invitrogen), following the manufacturer’s protocol. The transfection complexes were prepared in Opti-MEM, where solution A consisted of 15 μL of Lipofectamine 3000 mixed with 380 μL of Opti-MEM, and solution B contained 50 nM siRNA combined with 190 μL of Opti-MEM. These solutions were incubated for 15 minutes before being mixed and applied to the cells. After a duration of 6 hours, the medium was replaced with complete culture medium. The efficiency of the knockdown was subsequently assessed via Western blot analysis conducted 48 hours post-transfection.

### 2.16 Western blot

At the indicated time points (typically 48 h post-transfection), total protein was extracted using ice-cold RIPA buffer supplemented with protease and phosphatase inhibitors. After PBS wash, cells were lysed on ice for 20 min and clarified by centrifugation (14,000 rpm, 15 min, 4°C). Protein concentration was determined by BCA assay. Equal protein (30 µg) was mixed with 5× loading buffer, denatured at 100°C for 5 min, resolved by SDS-PAGE, and transferred to PVDF membranes using a semi-dry apparatus (≈300 mA, ~30 min; gel-dependent). Membranes were blocked in 5% non-fat milk (TBST) for 1 h at room temperature and incubated overnight (4°C) with primary antibodies: anti-TREX1 and anti-GAPDH. After TBST washes, HRP-conjugated secondary antibodies were applied for 2 h at room temperature. Signals were developed with ECL and imaged; band intensities were quantified in ImageJ and normalized to GAPDH.

### 2.17 EdU incorporation assay

Cells were initially seeded in suitable culture vessels and allowed to reach approximately 70% confluence. Subsequently, EdU was introduced to the culture medium at a concentration of 10 μM and incubated for a duration of 1 to 2 hours at 37°C. Post-incubation, the cells were fixed using 4% paraformaldehyde for 15 minutes at room temperature, followed by permeabilization with 0.5% Triton X-100 in phosphate-buffered saline (PBS) for 20 minutes. A Click-iT^®^ reaction cocktail was then applied in accordance with the manufacturer’s instructions. After thorough washing, the nuclei were counterstained with Hoechst 33342 at a concentration of 2 μg/mL for 5 to 10 minutes, ensuring protection from light. Finally, the cells were visualized using fluorescence microscopy at the appropriate wavelengths.

### 2.18 Transwell migration and invasion assays

For the migration assay, cells were trypsinized and resuspended in serum-free medium 24 hours post-transfection. A total of 2×10^4 cells in 200 μL were introduced into the upper chamber of a Transwell insert (8 μm pore size, devoid of Matrigel). The lower chamber was supplemented with 600 μL of medium containing 20% fetal bovine serum (FBS). Following a 24-hour incubation period, non-migrated cells were eliminated from the upper surface, while the migrated cells adhering to the lower membrane were fixed using 4% paraformaldehyde for 30 minutes. Subsequently, these cells were stained with 0.1% crystal violet for an additional 30 minutes and counted in five randomly selected fields using a microscope.

For invasion, inserts were pre-coated with Matrigel (300 μL of 1:6 diluted Matrigel, incubated at 37°C for 4 h to form a gel). 5×10^4 cells in 200 μL serum-free medium were added to the upper chamber; the lower chamber contained 600 μL medium with 20% FBS. After 24 h, invasion assays proceeded as above (fixation, staining, counting in five fields).

### 2.19 Statistical analysis

All experiments were conducted with a minimum of three replicates. The data are expressed as mean ± standard deviation. Group differences were evaluated using either Student’s t-test or Chi-square test, as deemed appropriate. Survival curves were analyzed through the log-rank test. Correlation analyses were performed using Spearman’s rank correlation coefficient. A two-sided P-value of less than 0.05 was regarded as statistically significant. Statistical analyses were carried out utilizing R software (version 4.2.0) and GraphPad Prism 9.

## 3 Results

### 3.1 Identification of immunogenic cell death/ferroptosis-related genes and differential expression

**Figure 1:**
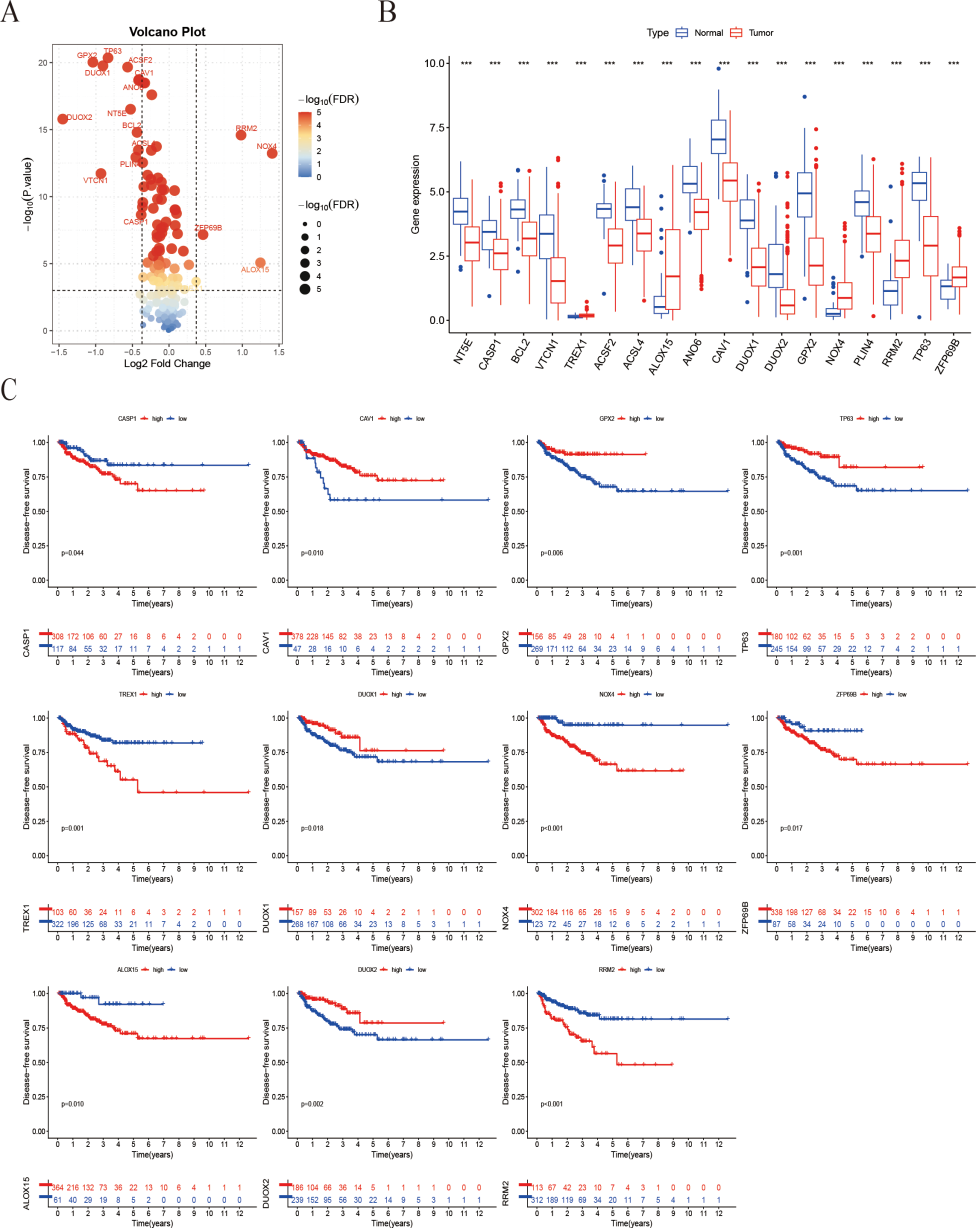
Screening of IFRGs and prognostic analysis. **(A)** The volcano plot shows the significance distribution of differentially expressed genes. **(B)** The box plot illustrates the differences in gene expression between tumor and normal tissues (“***” indicates p < 0.001). **(C)** Kaplan-Meier survival curve analysis of patient Progression-free survival stratified by IFRG expression.

A comprehensive multi-step screening approach was employed to identify genes that are associated with both ICD and ferroptosis, which exhibit differential expression in prostate cancer. Utilizing data from TCGA-PRAD, Pearson correlation analysis was performed between 44 ICD-related genes and 259 ferroptosis-related genes, resulting in the identification of 219 genes with significant correlations (|R| > 0.4, P < 0.05). Through further screening, a total of 18 genes were identified with significant expression changes based on the criteria of p-value < 0.001 and fold-change > 1.3 and boxplots were generated to demonstrate their pronounced expression differences (**Figures 1A, B**). Kaplan-Meier survival analysis indicated that 11 of the identified IFRGs were significantly correlated with PFS (**Figure 1C**), indicating that each of these genes has prognostic value on its own and justifying their inclusion in the model (**Figure 1C**). Notably, high expression levels of CAV1, GPX2, TP63, DUOX1, and DUOX2 were associated with improved prognosis, while elevated expression of CASP1, TREX1, NOX4, ZFP69B, ALOX15, and RRM2 was linked to poorer outcomes. Among these, NOX4, RRM2, and TREX1 exhibited particularly strong associations (P < 0.001).

### 3.2 Mutation, copy-number variation, and expression analysis of IFRGs in prostate cancer

**Figure 2:**
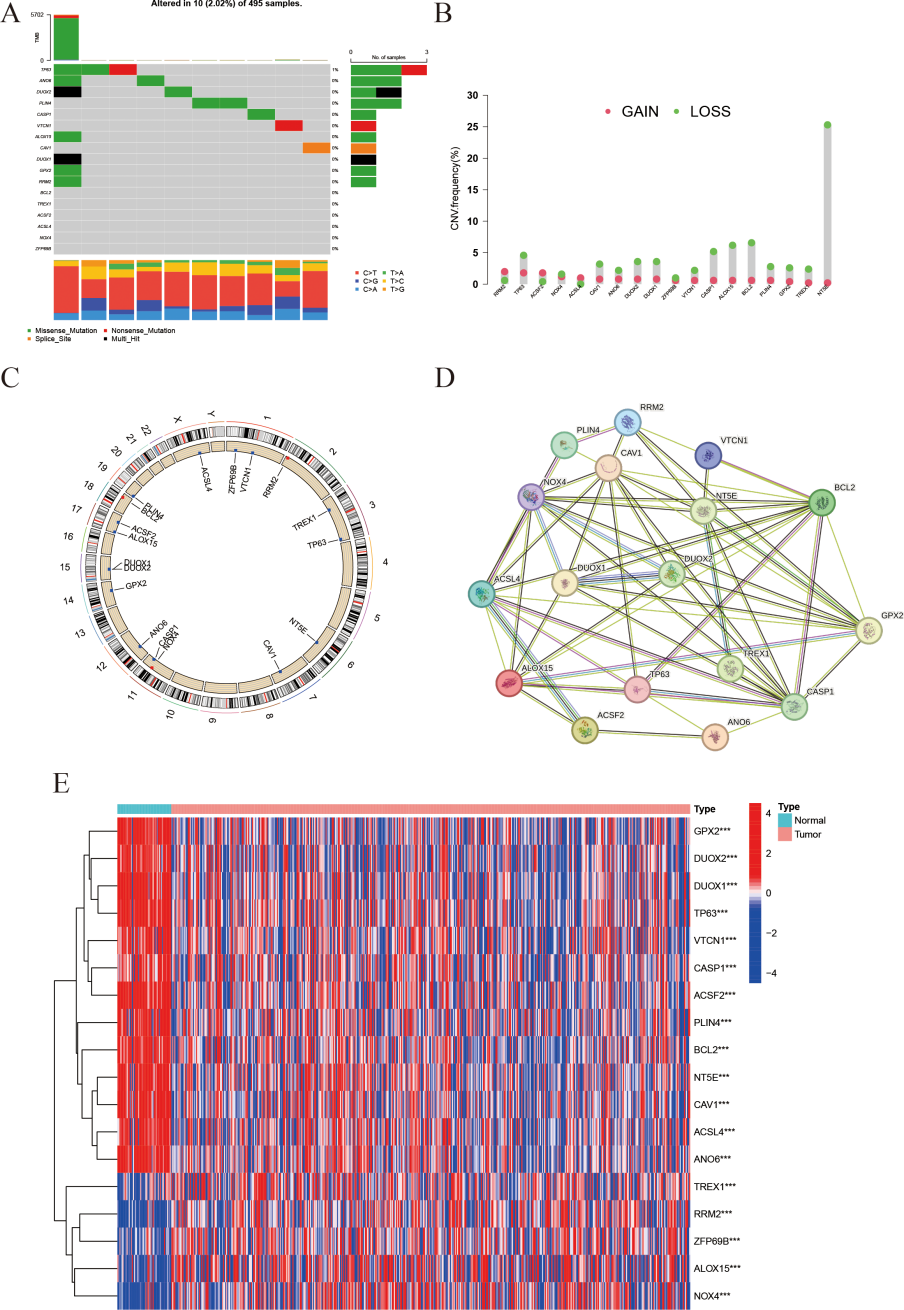
Landscape of IFRGs: mutation spectrum, copy-number variations, and expression profiles. **(A)** The waterfall plot presents the mutation types and frequencies of genes across samples. **(B)** The bar chart represents copy number variations (CNVs), with red dots indicating copy-number gains and green dots indicating losses. **(C)** The Circos plot shows the chromosomal distribution of the studied genes. **(D)** The protein-protein interaction (PPI) network illustrates interactions among the genes, with line color indicating interaction type and line thickness indicating interaction strength. **(E)** The heatmap displays gene expression levels in tumor vs. normal tissues, with color intensity representing expression level (*** indicates p < 0.001).

The mutational profile of the 18 IFRGs was analyzed in a cohort of 495 samples from the TCGA-PRAD database. Among these samples, 10 (2.02%) exhibited mutations in the IFRGs, with a predominance of missense mutations, alongside occasional occurrences of nonsense and splice-site mutations. Notably, TP63 and DUOX2 exhibited the highest mutation frequencies (**Figure 2A**). Additionally, genes such as TP63, CAV1, NT5E, TREX1, and GPX2 demonstrated significant copy number variation (CNV) deletions, while all 18 IFRGs displayed CNV across various chromosomes (**Figure 2B**). The analysis revealed varying degrees of DNA copy number variation among these IFRGs, which were distributed throughout the genome without evident clustering (**Figure 2C**). A protein-protein interaction (PPI) network, constructed using the STRING database, indicated potential cooperative functions among these genes, suggesting their involvement in various biological processes, including signaling pathways, oxidative stress response, lipid metabolism, and apoptosis (**Figure 2D**). Furthermore, heatmap analysis revealed that TREX1 and RRM2 expression levels were significantly higher in tumor tissues compared to normal tissues, whereas GPX2, DUOX1, and DUOX2 were expressed at lower levels in tumor tissues (**Figure 2E**). These findings may imply a potential relationship between IFRGs and the pathogenesis of prostate cancer.

### 3.3 Unsupervised clustering and survival analysis

**Figure 3:**
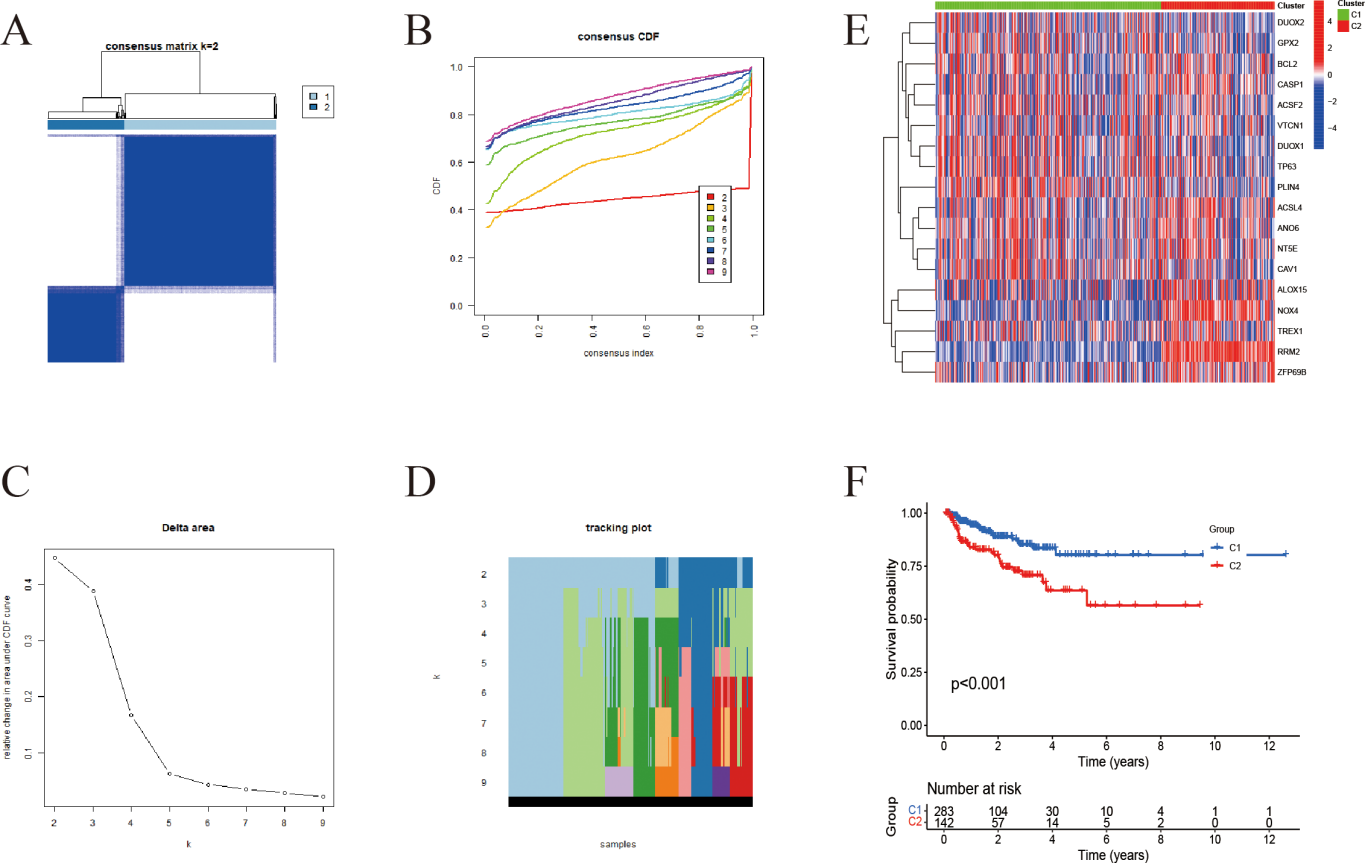
Unsupervised clustering-based subtyping using IFRGs. **(A)** Consensus clustering matrix for k=2; darker blue squares indicate higher similarity between samples. **(B)** Cumulative distribution function (CDF) curves, with different colors representing different k values. **(C)** Tracking plot showing how sample classifications change with varying k **(D)** Delta area plot used to assess changes in clustering stability across different k values. **(E)** Heatmap displaying the expression patterns of immunogenic cell death- and ferroptosis-related genes across the two subtypes. **(F)** Kaplan-Meier survival curves for C1 and C2 subgroups.

Using expression data from 18 IFRGs, consensus clustering was conducted on a cohort of 549 samples obtained from the TCGA-PRAD database. This analysis successfully categorized the 549 patients into two distinct subgroups, designated as C1 and C2 (**Figures 3A–D**). Subsequent heatmap analysis revealed that the expression levels of IFRGs were significantly elevated in the C2 subtype (**Figure 3E**). Additionally, Kaplan-Meier survival analysis (**Figure 3F**) indicated that patients classified in subtype C2 experienced significantly poorer survival outcomes compared to those in subtype C1, with a statistically significant difference (P<0.01).

### 3.4 Immune microenvironment analysis

**Figure 4:**
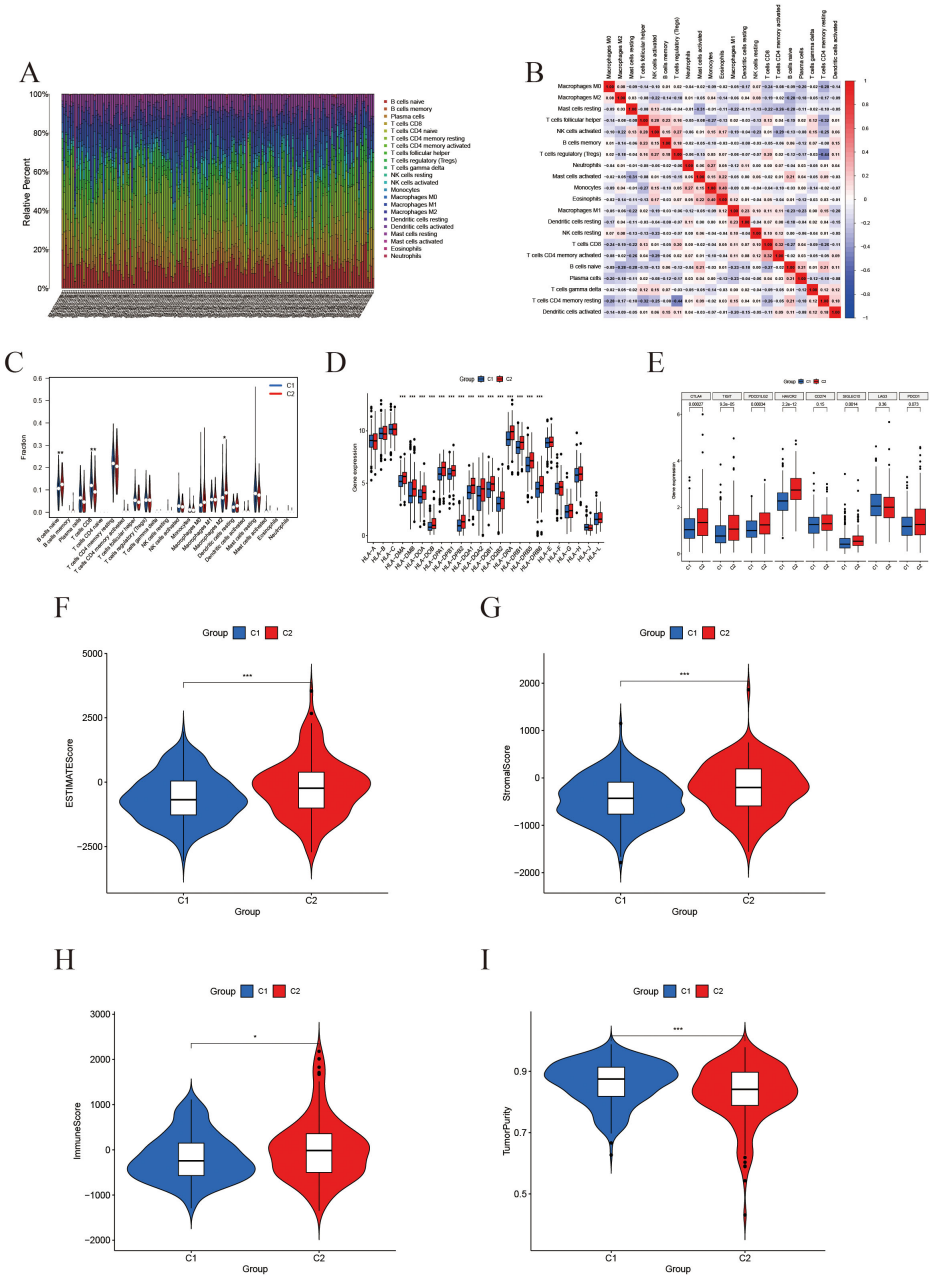
Immune microenvironment characterization based on IFRG subtypes. **(A)** Immune cell composition: A stacked bar plot of the relative fractions of 22 immune cell types in each sample, as estimated by the CIBERSORT algorithm. **(B)** Immune cell correlation heatmap: Spearman correlation coefficients among the infiltration levels of the 22 immune cell types; color intensity denotes the strength of correlation. **(C)** Violin plots comparing immune cell infiltration between the C2 group (red) and C1 group (blue). *, **, and *** indicate p < 0.05, 0.01, and 0.001, respectively. **(D)** Boxplot of Differential Expression of HLA Genes. **(E)** Boxplot of Differential Expression of Immune Checkpoints. **(F-I)** ESTIMATE score comparisons (violin plots): Comparison of the ESTIMATE composite score, StromalScore, ImmuneScore, and tumor purity between the two groups. The high-expression group shows significantly higher ESTIMATE and stromal scores, a modest increase in ImmuneScore, and a marked reduction in tumor purity.

CIBERSORT was employed to assess the infiltration levels of 22 distinct immune cell types. The results were illustrated through bar plots (**Figure 4A**) and correlation heatmaps (**Figure 4B**), which depicted the distribution of immune cells and their interrelations. Furthermore, validation using the EPIC and MCP-counter methods demonstrated that the overall immune landscape and major trends remained consistent (**Supplementary Figure 1**). Notable differences in immune cell populations were observed, particularly in CD8+ T cells, M2 macrophages, and regulatory T cells (**Figure 4C**). Additionally, gene expression profiles for HLA (**Figure 4D**) and immune checkpoint genes (**Figure 4E**) revealed significant variations between subtypes. The ESTIMATE analysis further demonstrated that the high-IFRG group had elevated immune and stromal scores, accompanied by reduced tumor purity (**Figures 4F–I**).

### 3.5 Differential expression and enrichment analyses of IFRG subtypes

**Figure 5:**
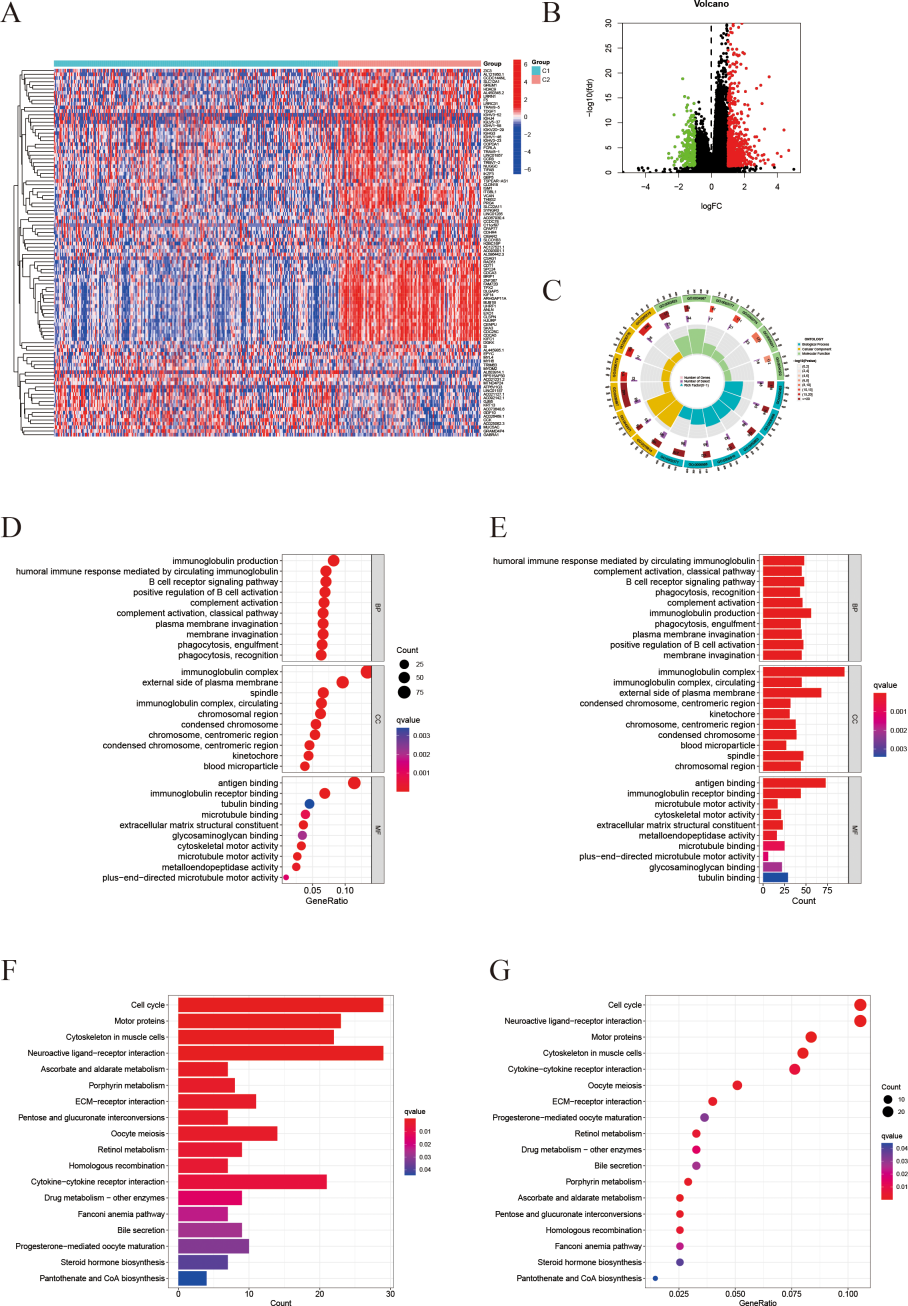
Differential gene expression profiling and enrichment analyses. **(A)** Heatmap of differentially expressed genes (DEGs) comparing the C2 and C1 groups. **(B)** Volcano plot visualizing the DEGs (threshold: |log_2_FC| > 1, P < 0.05). **(C)** Circos plot illustrating the genomic locations of the DEGs. **(D, E)** Gene Ontology (GO) enrichment analysis results for Biological Process (BP), Cellular Component (CC), and Molecular Function (MF) in the C2 group **(D)** and C1 group **(E)**. Redder color indicates a smaller q-value (higher enrichment significance). **(F, G)** KEGG pathway analysis: **(F)** shows the distribution of genes in significantly enriched pathways; **(G)** shows a bubble chart of enriched pathways, where dot size represents gene count and color indicates the q-value.

The “limma” package was employed to identify differentially expressed genes (DEGs) between the two subtypes, resulting in the identification of 1,032 DEGs (**Figure 5B**). Following this, the study focused on selecting significantly differential genes and created corresponding heatmaps (**Figure 5A**). Subsequently, Gene Ontology (GO) and Kyoto Encyclopedia of Genes and Genomes (KEGG) analyses were conducted to investigate the molecular mechanisms associated with differentially expressed transcripts related to ICD and ferroptosis (**Figures 5C–G**). The findings from the GO enrichment analysis revealed that immune-related processes, including humoral immune response, complement activation, B cell receptor signaling pathway, and phagocytosis, were the predominant enriched biological processes (BP). In terms of cellular components (CC), the primary enriched items included immune complexes, chromosomes, kinetochores, and platelet microparticles. Regarding molecular functions (MF), the major enrichments encompassed antigen binding, immunoglobulin receptor binding, and activities related to microtubules (**Figures 5C–E**). The KEGG pathway analysis highlighted tumor-related processes such as cell cycle regulation, cytoskeletal movement, immune signal transduction, and metabolic regulation as the key enriched factors (**Figures 5F, G**).

**Figure 6:**
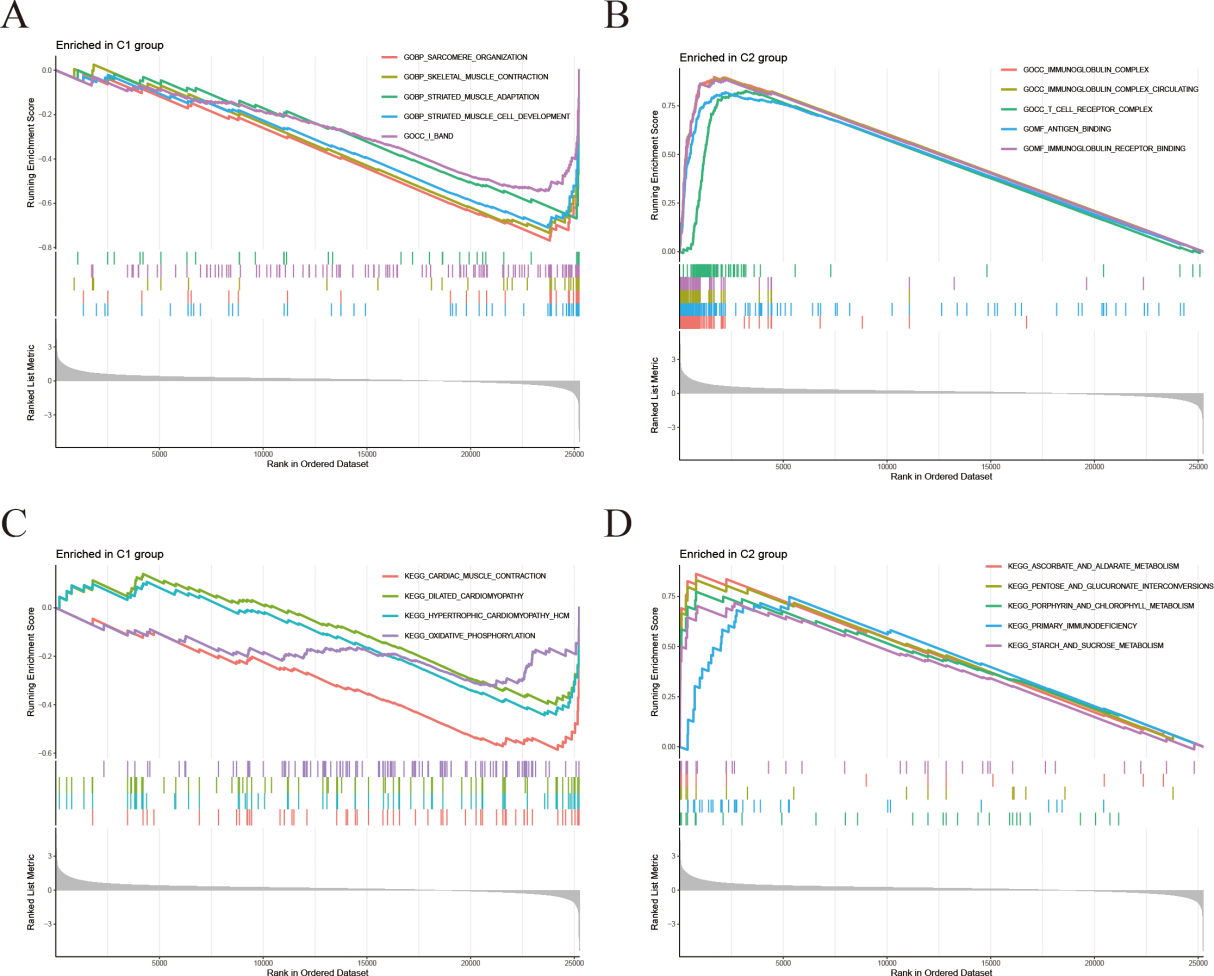
Differential gene GSEA analyses. **(A, B)** Gene Set Enrichment Analysis (GSEA): **(A)** Gene sets significantly enriched in C1 group; **(B)** Gene sets significantly enriched in C2 group. **(C, D)** KEGG-GSEA: **(C)** KEGG pathways enriched in C1 group; **(D)** KEGG pathways enriched in C2 group.

To further investigate the molecular mechanisms associated with the signature derived from IFRGs, we conducted Gene Set Enrichment Analysis (GSEA), as illustrated in **Figures 6A–D**. The functional enrichment analysis (**Figures 6A, B**) revealed that the C2 group had significant enrichment in pathways related to immune response, antigen recognition, and T-cell receptor signaling. Conversely, C1 group exhibited elevated expression of genes associated with the muscular system. In the KEGG pathway enrichment analysis (**Figures 6C, D**), C2 group was found to be enriched in immune-related pathways, as well as those involved in the cell cycle and cell adhesion molecules. In contrast, C1 group showed enrichment in pathways related to myocardial contraction and oxidative phosphorylation.

### 3.6 Construction of the IFRG-based prognostic model

**Figure 7:**
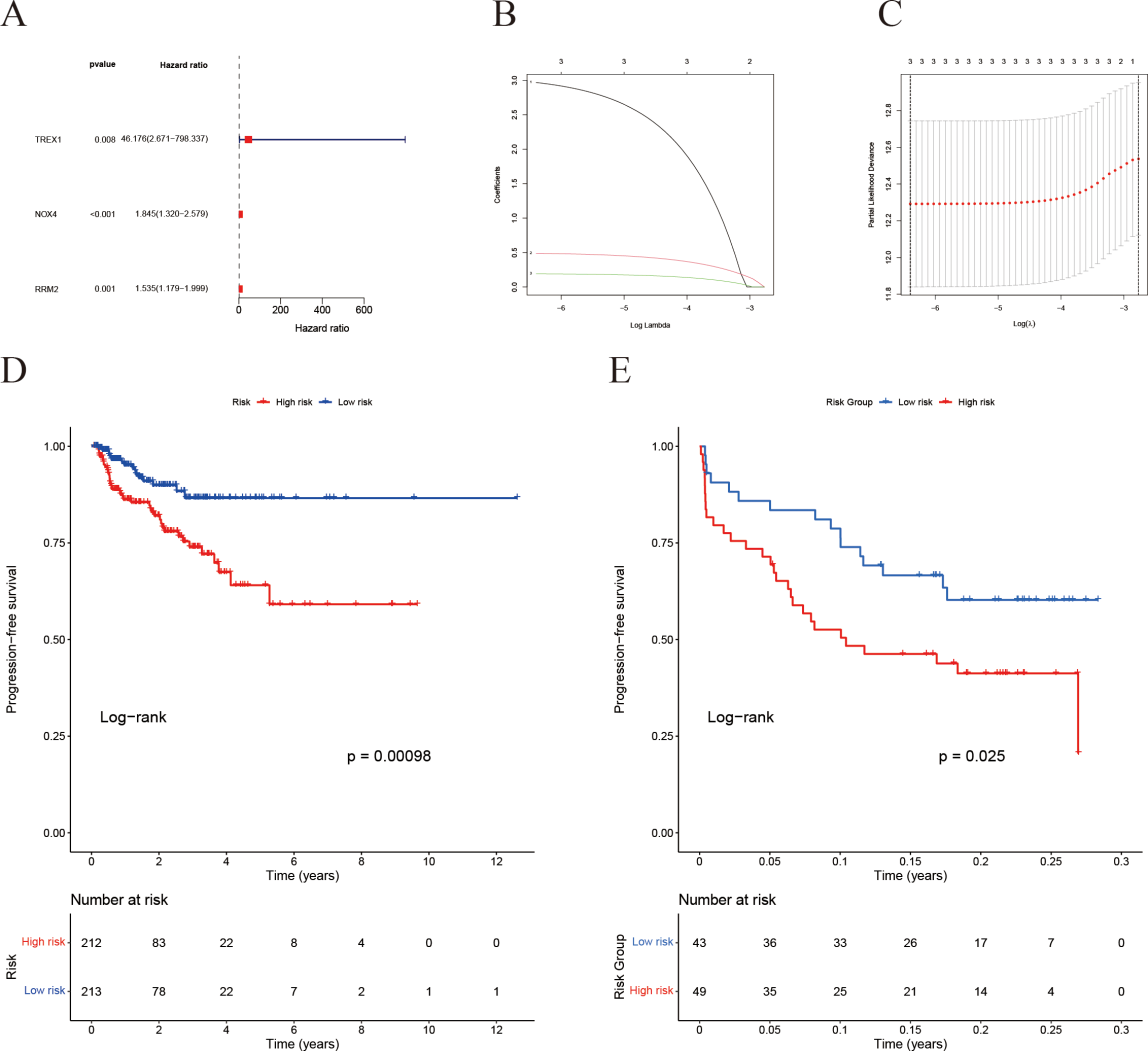
Construction of the IFRG-based prognostic model and survival analysis. **(A)** Univariate Cox regression analysis results. **(B)** LASSO regression cross-validation error curve: the y-axis represents the partial likelihood deviance. **(C)** LASSO regression coefficient trajectory: different colored curves represent different genes, and as log(λ) increases, the gene coefficients shrink toward zero—ultimately identifying the key genes for the model. **(D)** Kaplan-Meier survival analysis for the TCGA cohort. **(E)** Kaplan-Meier survival analysis for the GEO validation cohort.


A LASSO Cox regression analysis was conducted to develop a prognostic signature utilizing the previously mentioned 18 IFRGs. This analysis identified three specific IFRGs: TREX1, NOX4, and RRM2 (**Figure 7A**). Cross-validation techniques were employed to ascertain the optimal penalty coefficient (λ.min) for the LASSO regression, with the corresponding LASSO path diagram and cross-validation curve illustrated in **Figures 7B, C**. The risk score was computed using the following formula:

$$\text{Risk score}=\left(2.9692\times \text{TREX}1\right)+\left(0.4886\times \text{NOX}4\right)+\left(0.1914\times \text{RRM}2\right)$$

Subsequently, patients were categorized into high-risk and low-risk groups based on the median risk scores. Kaplan-Meier survival analysis revealed that the high-risk cohort exhibited a significantly poorer prognosis in both the TCGA (**Figure 7D**) and GEO (**Figure 7E**) datasets.

### 3.7 Validation of the prognostic model and nomogram construction

**Figure 8:**
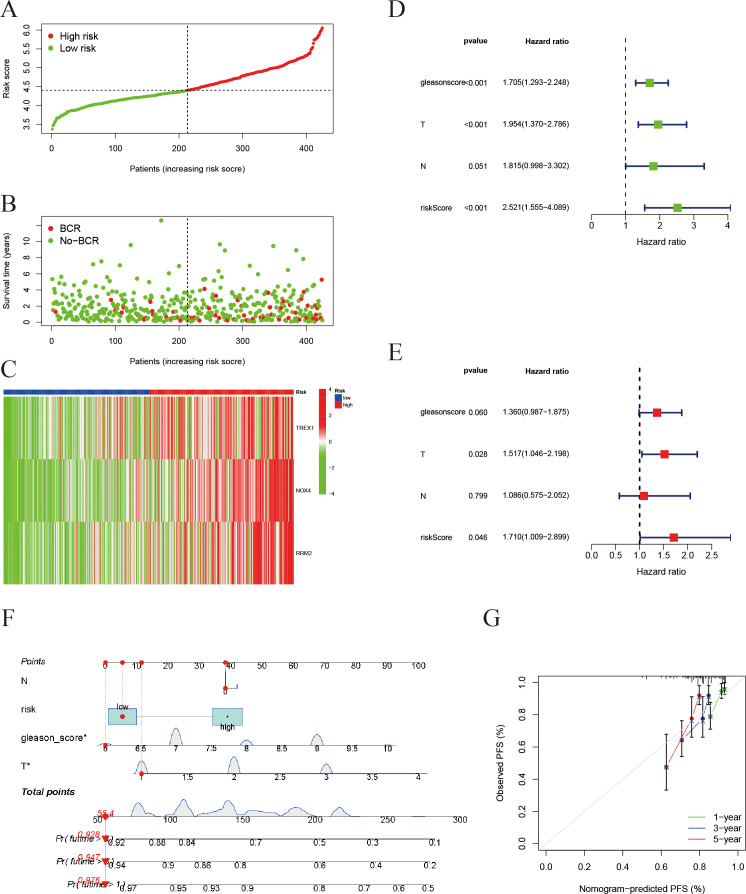
Validation of the risk score model and prognostic evaluation. **(A)** Risk score distribution: Patients are ranked by risk score and divided into a high-risk group (red) and a low-risk group (green). The x-axis represents the sorted patients (low to high risk) and the y-axis is the risk score. **(B)** Survival status plot for prostate cancer PFS: Patients are ordered by their risk score on the x-axis, and progression-free survival time (in years) is on the y-axis. Red dots represent patients who experienced progression, while green dots represent those who remain progression-free. **(C)** Heatmap of gene expression: Shows expression of immunogenic cell death/ferroptosis-related genes (TREX1, NOX4, RRM2) in the high- and low-risk groups (red = high, green = low) (In panels A-C, patients on the x-axis are ordered by increasing risk score). **(D)** Univariate Cox regression analysis (forest plot): The impact of different clinical factors (Gleason score, tumor stage T, lymph node status N, and risk score) on prognosis. The x-axis is the hazard ratio (HR); green squares denote HR < 1 (protective factors) and blue squares denote HR > 1 (risk factors). **(E)** Multivariate Cox regression analysis: The risk score remains an independent prognostic factor (HR = 1.71, P = 0.046). **(F)** Construction of a prognostic nomogram for estimating the 1-, 3-, and 5-year progression-free survival probabilities in patients with prostate cancer. **(G)** Calibration plots assessing the agreement between nomogram-predicted and observed 1-, 3-, and 5-year progression-free survival outcomes.

Risk stratification analysis revealed that patients in the high-risk group exhibited a markedly higher incidence of biochemical recurrence compared with those in the low-risk group (**Figures 8A, B**). The gene expression heatmap indicated a significant upregulation of TREX1, NOX4, and RRM2 in the high-risk group relative to the low-risk group (**Figure 8C**). Univariate analysis established a significant correlation between the risk score, Gleason score, and T stage with PFS in prostate cancer patients (all P < 0.001) (**Figure 8D**). Furthermore, multivariate Cox regression analysis validated that both the risk score and T stage independently influenced patient prognosis (P = 0.046 and P < 0.001, respectively) (**Figure 8E**). Subsequently, an individualized prognostic model was formulated in the form of a nomogram (**Figures 8F, G**), which demonstrated strong predictive accuracy for estimating PFS at 1, 3, and 5 years. The calibration plots indicated good concordance between the predicted and observed outcomes at 1 and 3 years, while a noticeable deviation was observed at 5 years, possibly due to the limited follow-up duration and the influence of competing risks.

### 3.8 Correlation of risk score with immune infiltration and drug sensitivity

**Figure 9:**
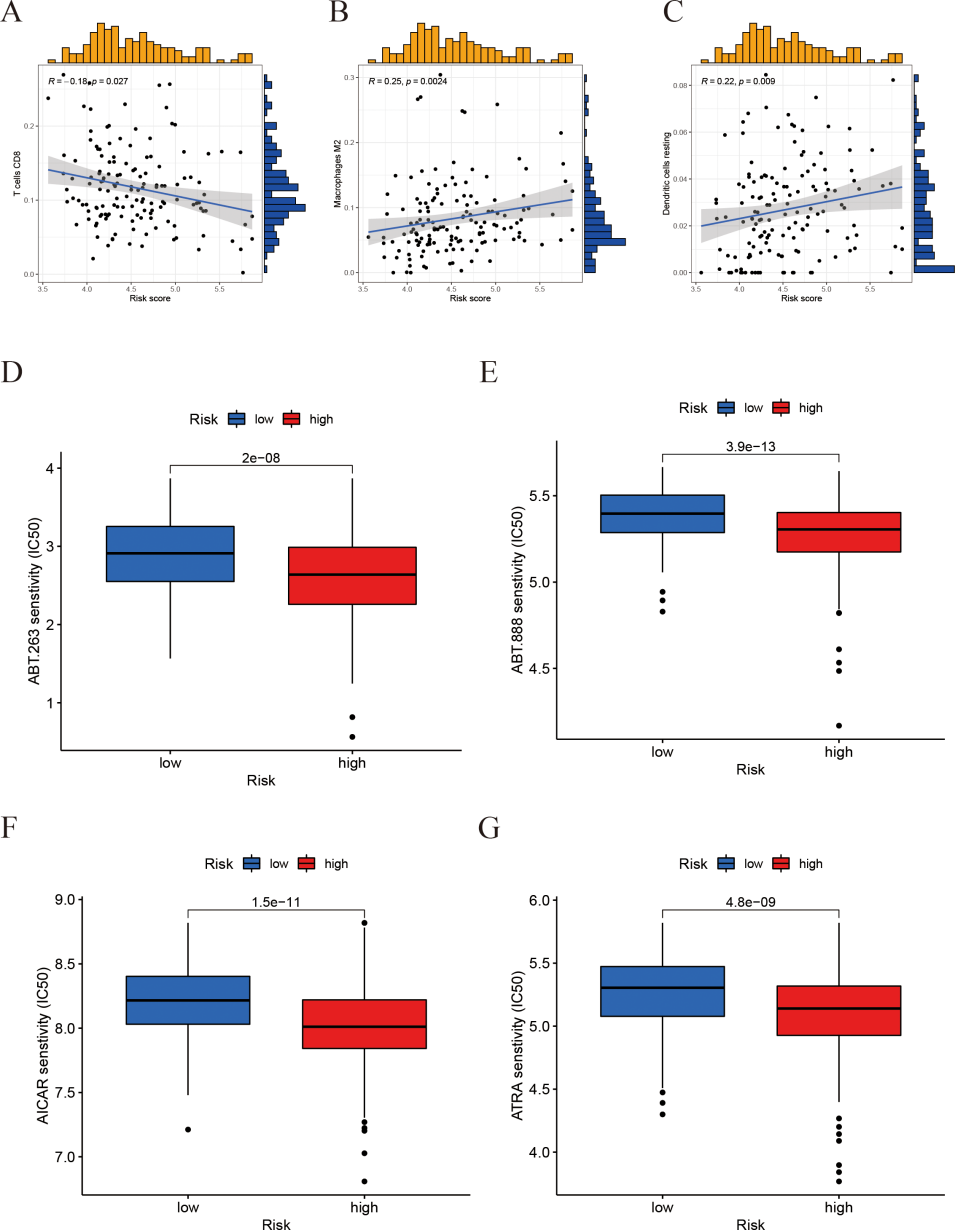
Correlation of the risk score with immune‐cell infiltration and drug sensitivity. **(A-C)** Correlation between the risk score and immune cell infiltration levels (scatter plots for different immune cell types). **(D-G)** Drug sensitivity: A box plot comparing the half-maximal inhibitory concentration (IC50) in the high-risk vs. low-risk groups.

To further clarify the immunological implications associated with the prognostic model, we performed an analysis of immune cell correlations. The findings indicated a negative correlation between the risk score and the presence of CD8^+^ T cells (**Figure 9A**). Conversely, M2-type macrophages and resting dendritic cells exhibited a positive correlation with the risk score (**Figures 9B, C**). These results imply that individuals with elevated risk scores may experience some immune suppression or dysfunction, evidenced by fewer CD8^+^ T cells. Furthermore, the drug sensitivity analysis (**Figures 9D–G**) revealed that the high-risk group demonstrated significantly reduced half-maximal inhibitory concentrations (IC_50_) for ABT-263, ABT-888, AICAR, and ATRA.

### 3.9 Risk score and somatic mutation/tumor mutation burden

**Figure 10:**
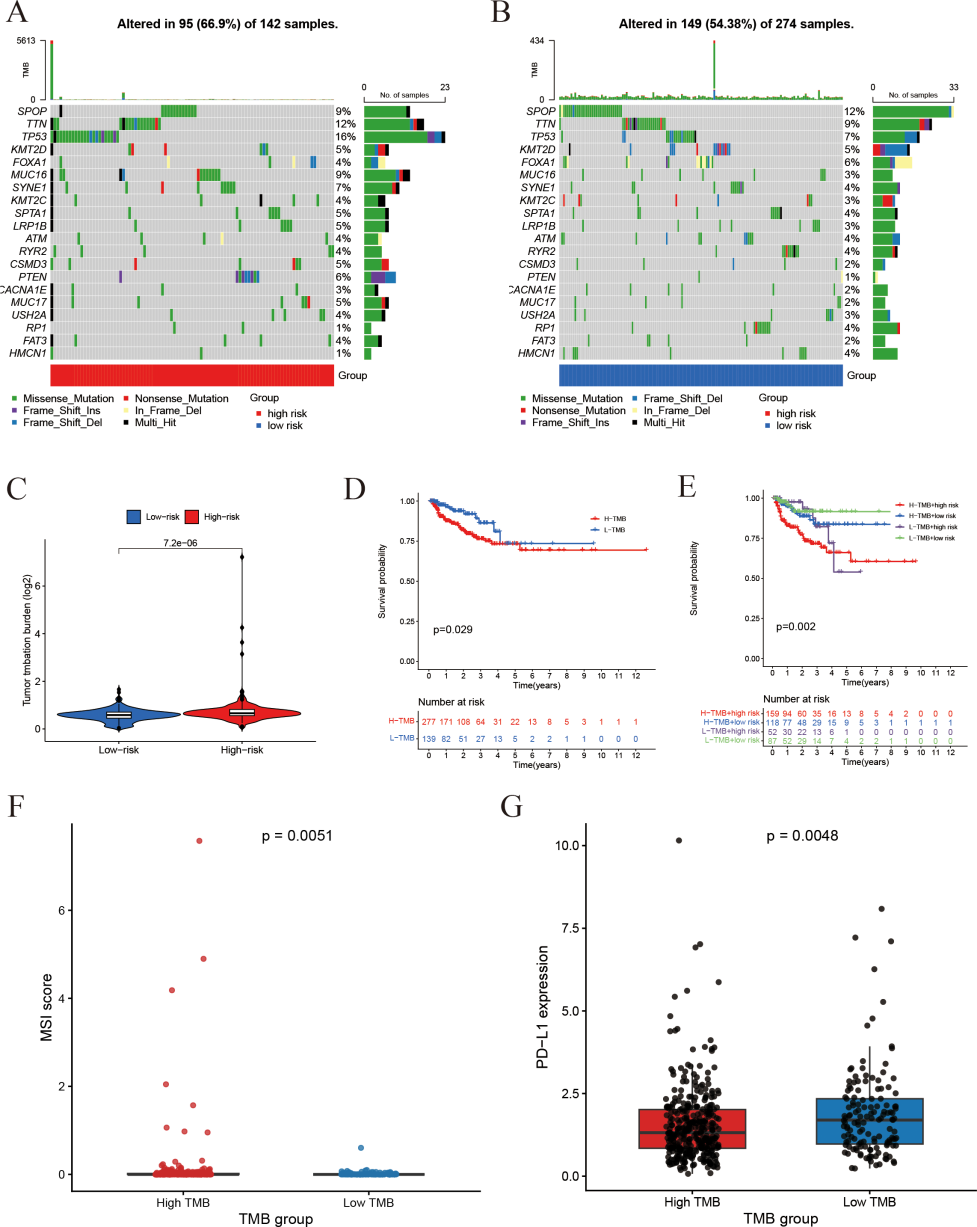
Association of risk score with somatic mutations and tumor mutation burden. **(A, B)** Somatic mutation profiles: **(A)** Gene mutation profile of high-risk group; **(B)** Gene mutation profile of the low-risk group. **(C)** Tumor mutation burden (TMB) comparison between groups. **(D)** Impact of TMB on Progression-free survival. **(E)** Combined effect of TMB status and risk score on Progression-free survival. **(F)** Comparison of MSI scores between the high-TMB and low-TMB groups. **(G)** Comparison of PD-L1 expression between the high-TMB and low-TMB groups.

To gain additional insights into the reliability of the prognostic model, we conducted an analysis of the somatic mutation landscape. The mutation plots indicate that the four most frequently mutated genes in the high-risk cohort (**Figure 10A**) were SPOP, TTN, TP53, and KMT2D, which were also the predominant mutated genes in the low-risk cohort (**Figure 10B**). This observation suggests that there is no significant disparity in somatic mutation patterns between the high- and low-risk groups. Regarding tumor mutation burden (TMB) (**Figure 10C**), the high-risk group demonstrated a markedly elevated TMB. Kaplan-Meier survival analyses (**Figures 10D, E**) illustrated that both the risk score and TMB levels were significantly correlated with patient prognosis, with lower TMB and risk scores associated with improved survival outcomes. TMB levels were positively correlated with microsatellite instability (MSI) and PD-L1 expression (**Figures 10F, G**), suggesting that increased mutational load may contribute to enhanced immunogenicity and immune checkpoint activation in prostate cancer.

### 3.10 TREX1-associated tumor microenvironment alterations

**Figure 11:**
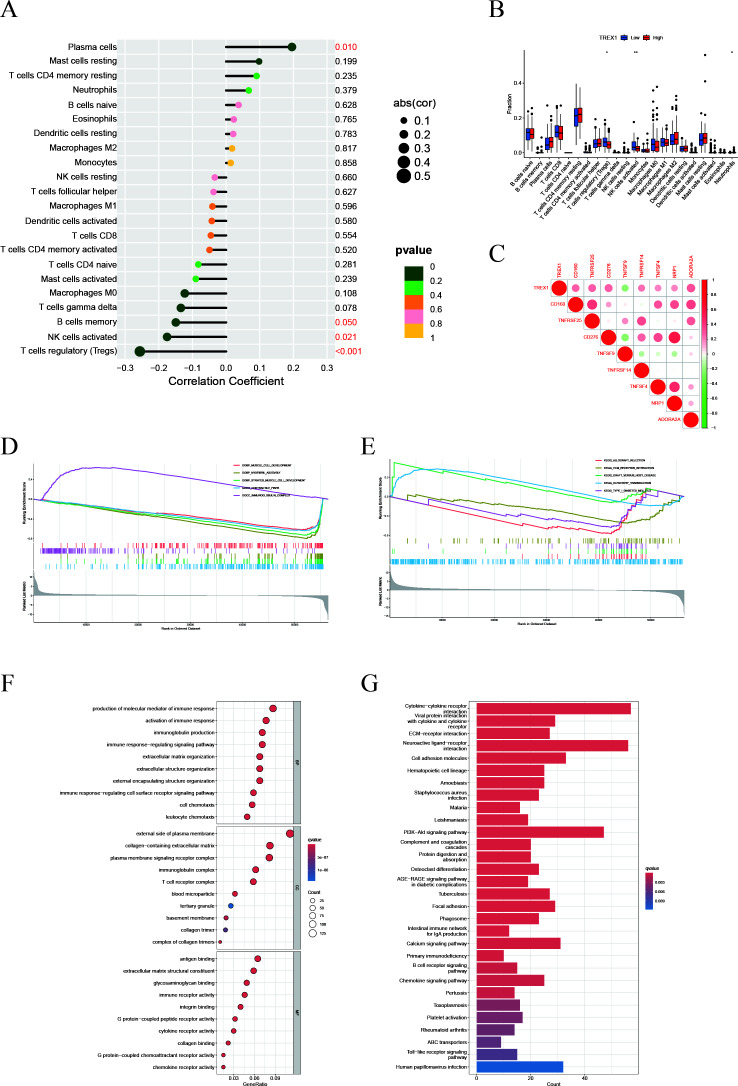
Immune infiltration and functional enrichment analyses related to TREX1 in prostate cancer. **(A)** Correlation between TREX1 expression and 22 immune cell types. **(B)** Immune cell abundance comparison between high and low TREX1 expression groups. **(C)** Correlation of TREX1 with key immune checkpoint genes. **(D, E)** GSEA showing immune-related pathway enrichment in TREX1 high vs. low groups. **(F)** GO enrichment bubble plot of TREX1. **(G)** KEGG pathway enrichment bar plot of TREX1.

Within the IFRG model, TREX1 showed the highest coefficient, underscoring its potential importance in prostate cancer progression. Additional analyses focusing on its immunological role revealed that TREX1 expression was negatively correlated with the infiltration of activated NK cells and regulatory T cells, while showing a modest positive correlation with plasma cells (**Figures 11A, B**). Moreover, TREX1 expression was positively associated with several immune checkpoint genes, including CD160, TNFRSF25, TNFSF9, and ADORA2A, suggesting potential co-expression patterns relevant to immune regulation (**Figure 11C**).

Gene set enrichment analysis (GSEA) further indicated that tumors with lower TREX1 expression were enriched in immune activation pathways, such as positive regulation of immune response and lymphocyte migration, whereas tumors with higher TREX1 expression were enriched in pathways related to cell cycle progression, extracellular matrix organization, and collagen metabolism (**Figures 11D, E**). Complementary enrichment analyses supported these findings, with GO analysis (**Figure 11F**) highlighting extracellular matrix-related processes, including structural organization and collagen binding, and KEGG analysis (**Figure 11G**) showing enrichment in pathways related to extracellular matrix remodeling, cell adhesion, and cell cycle regulation. Collectively, these results suggest that TREX1 expression is closely linked to changes in immune activity, proliferative capacity, and extracellular matrix dynamics within the tumor microenvironment.

### 3.11 TREX1 expression in prostate cancer tissues

**Figure 12:**
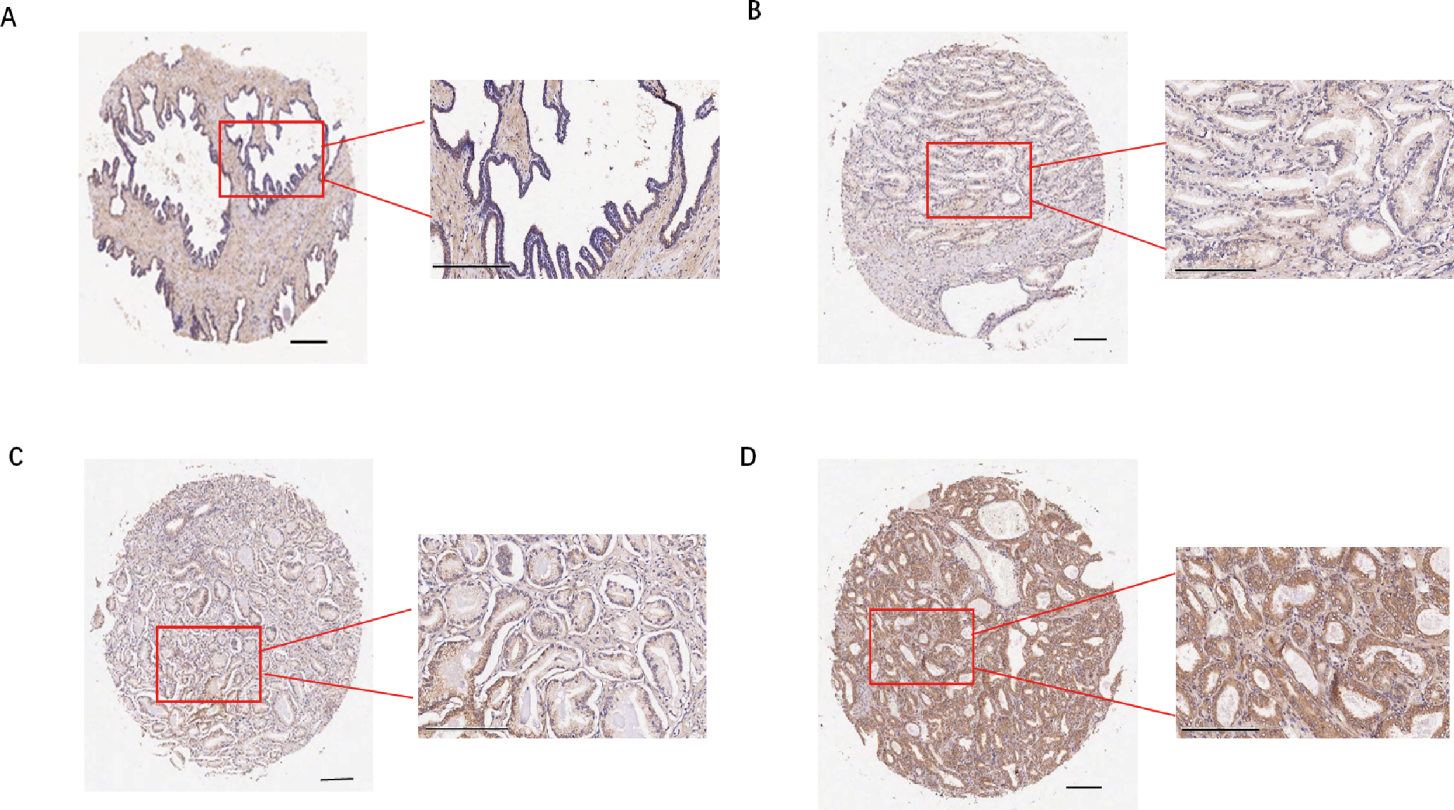
Immunohistochemistry and cellular function analysis. **(A-D)** Representative images of staining results **(A)** Adjacent normal tissue, negative; **(B)** Cancer tissue, weak positive (+); **(C)** Cancer tissue, moderate positive (++); **(D)** Cancer tissue, strong(+++).

Subsequently, we examined the expression of the TREX1 protein in clinical specimens. Immunohistochemical (IHC) analysis was conducted on a tissue microarray comprising 60 prostate cancer samples and 57 corresponding adjacent non-tumor tissues (**Figures 12A–D**). The IHC results revealed that TREX1 protein was localized in the cytoplasm of tumor cells, as indicated by brown-yellow staining, which was considered positive, whereas blue staining denoted negative expression. Based on staining intensity and distribution, prostate tissue samples were stratified into two groups: low expression (scores 0-3) and high expression (scores 4-9). Among the prostate cancer specimens, 45 cases (75%) exhibited high TREX1 expression, while 15 cases (25%) showed low expression. Conversely, in the adjacent normal tissues, 24 cases (42%) demonstrated high TREX1 expression, and 33 cases (58%) were classified as low expression (**Table 1**). Statistical analysis confirmed that the difference in TREX1 expression between tumor and adjacent tissues was significant (P = 0.0003) (**Table 2**).

**Table 1:** Differential expression of TREX1 in prostate cancer and adjacent normal tissues.

Tissue type	Low expression (0-3)	High expression (4-9)	Total
Tumor	15	45	60
Adjacent Normal	33	24	57
Total	48	69	117

**Table 2:** Differential expression statistics of TREX1 in prostate cancer and adjacent normal tissues.

Tissue type	Total (n)	TREX1 expression		χ² Value	P value
		High	Low		
Tumor	60	45	15	13.08	0.0003
Adjacent Normal	57	24	33		

### 3.12 Effect of TREX1 knockdown on proliferation, migration, and invasion of prostate cancer cells

**Figure 13:**
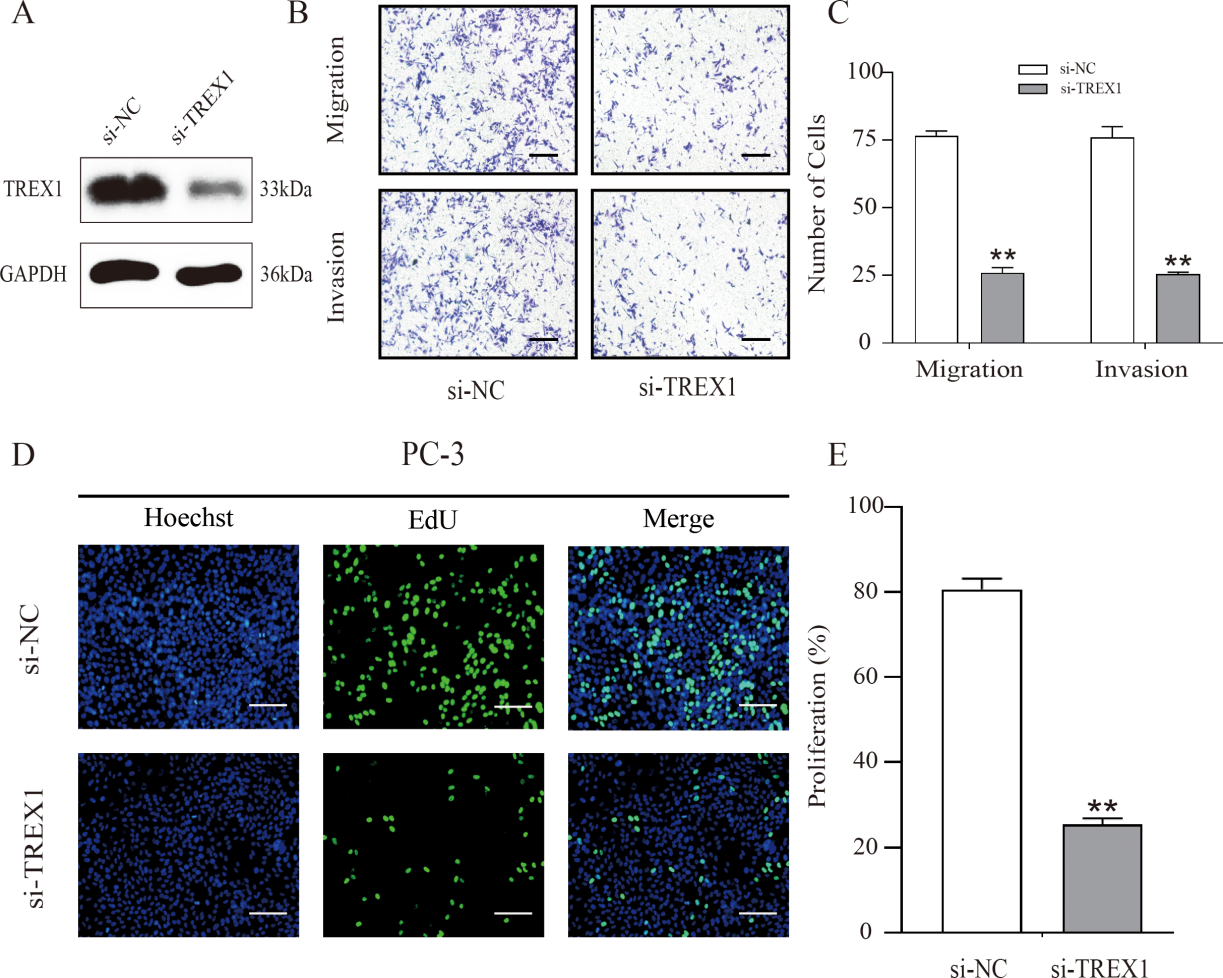
TREX1 knockdown suppresses proliferation, migration, and invasion of PC-3 cells. **(A)** Western blot analysis confirming efficient knockdown of TREX1 expression in PC-3 cells transfected with TREX1-specific siRNA (Si-TREX1) compared with negative control (Si-NC), with GAPDH as the loading control. **(B)** Representative images of Transwell migration and invasion assays showing reduced numbers of migrating and invading cells after TREX1 silencing. **(C)** Quantification of migrating and invading cells demonstrating a significant reduction in the si-TREX1 group compared to Si-NC. **P < 0.01.**(D)** EdU incorporation assay showing decreased numbers of proliferating cells in TREX1-knockdown cells. Hoechst (blue) stains nuclei, EdU (green) labels proliferating cells, and merged images are shown. **(E)** Quantification of proliferating cells indicating a significant reduction in the si-TREX1 group compared with controls. **P < 0.01.

To investigate the functional role of TREX1 in prostate cancer, small interfering RNA (siRNA)-mediated knockdown experiments were conducted utilizing PC-3 cells. Western blot analysis confirmed a marked reduction in TREX1 protein expression following transfection with TREX1-specific siRNA compared with negative control (Si-NC) (**Figure 13A**). Transwell assays demonstrated that TREX1 knockdown significantly suppressed both cell migration and invasion, with markedly fewer migrating and invading cells observed in the si-TREX1 group relative to Si-NC (P < 0.01) (**Figures 13B, C**). EdU incorporation assays further revealed a substantial decrease in proliferating cells in the si-TREX1 group compared to controls (P < 0.01) (**Figures 13D, E**). These findings indicate that TREX1 promotes prostate cancer progression by facilitating tumor cell proliferation, migration, and invasion.

## 4 Discussion

Prostate cancer is recognized as one of the most prevalent malignant neoplasms within the urinary system, exhibiting the highest incidence among male urogenital cancers, thereby posing a significant threat to public health (1). ICD and ferroptosis are two recently recognized forms of regulated cell death, and accumulating evidence indicates that both processes play crucial roles in the progression of various malignancies, including prostate cancer (23, 24). Furthermore, emerging evidence suggests that ferroptosis may alter the tumor immune microenvironment through the release of cytokines and DAMPs, which in turn can enhance immunogenic cell death. In cases of high-risk prostate cancer, sustained ferroptotic and inflammatory stress may facilitate the recruitment of immunosuppressive myeloid cells, such as M2-type macrophages, thereby promoting immune evasion (25, 26). This study undertakes a systematic examination of the transcriptional changes and interactions between gene sets associated with ICD and ferroptosis in prostate cancer, with the objective of elucidating their combined influence on tumor progression and immune regulation.

Our bioinformatics analysis identified 18 IFRGs through the intersection of ICD and ferroptosis gene networks. It is noteworthy that prior research has examined gene signatures associated with either immunogenic cell death or ferroptosis in the context of prostate cancer independently. For instance, Kang et al. established an ICD-related gene signature in prostate cancer that effectively stratified patients based on their prognosis (14), while Wang et al. developed a ferroptosis-associated risk model that demonstrated a correlation with the tumor immune microenvironment (13). In contrast, our study uniquely integrates both ICD and ferroptosis into a cohesive model, thereby reflecting the potential interactions between these two cell death pathways. We have combined ICD and ferroptosis to create a prognostic model for prostate cancer, utilizing differentially expressed IFRGs. This model demonstrated significant prognostic value, underscoring the clinical relevance of these biomarkers in the context of prostate cancer.

The clustering analysis based on the IFRG identified two distinct subtypes of prostate cancer, designated as C1 and C2, which exhibit significant disparities in survival outcomes. This finding suggests that the interaction between the pathways of ICD and ferroptosis may influence the aggressiveness of the disease. Subtype C2 is associated with a poorer prognosis and is characterized by an enrichment of immune-related pathways. This seemingly paradoxical characterization of C2 as an “immune-active” yet high-risk tumor subtype may be elucidated by the quality and context of the immune response. Our analyses of immune cell populations indicate that C2 tumors are infiltrated by elevated levels of M2 macrophages and other immunosuppressive components, which likely foster a tumor-promoting inflammatory milieu. Similar patterns have been observed in other malignancies (27–29) where an influx of tumor-associated macrophages and regulatory T cells correlates with accelerated tumor progression, even in the presence of a substantial number of T cells. In contrast, subtype C1 is characterized by a lower overall immune cell presence (immune-cold) but a relatively higher proportion of cytotoxic lymphocytes, which may contribute to more effective tumor control.

In order to address the variability among patients, we have created and validated an innovative prognostic model for prostate cancer that is grounded in genes associated with immunogenic cell death and ferroptosis. The risk score derived from this model demonstrated a strong correlation with patient outcomes, levels of immune checkpoint expression, and the extent of immune cell infiltration, underscoring its potential utility in characterizing the tumor immune landscape.

Moreover, drug sensitivity analysis derived from the current model indicates that patients classified within the high-risk group may exhibit increased susceptibility to specific therapeutic agents. Specifically, the BCL-2/BCL-XL inhibitor ABT-263 (Navitoclax) showed lower IC_50_ values, consistent with reports that inhibition of BCL-2 family proteins enhances apoptosis and chemosensitivity (30). The PARP inhibitor ABT-888 (Veliparib) showed a trend toward increased activity, with case reports and early-phase studies suggesting that tumors harboring homologous recombination repair deficiencies, particularly BRCA2 alterations, may be more susceptible; however, evidence in prostate cancer remains limited (31). Similarly, the AMPK activator AICAR exhibited greater potency, aligning with preclinical evidence that AICAR suppresses proliferation, induces apoptosis, and impairs metastasis via the AMPK/mTOR pathway (32). In addition, all-trans retinoic acid (ATRA) showed enhanced efficacy in high-risk cases, which may be linked to its ability to induce differentiation, apoptosis, and immune modulation (33). Collectively, these findings underscore that tumors classified as high-risk by the IFRG signature may possess distinct therapeutic susceptibilities. Further preclinical and clinical studies are warranted to validate these strategies.

Furthermore, our results demonstrate that the DNA exonuclease TREX1, a key element of our gene signature, is upregulated in prostate cancer tissues and exhibits pro-tumorigenic properties *in vitro*. TREX1, a 3’ to 5’ DNA exonuclease encoded by a gene located on human chromosome 3p21.31 (34, 35), is ubiquitously expressed and plays a vital role in degrading cytosolic DNA, thereby preventing inappropriate activation of the innate immune system (36). Impairment of TREX1 function leads to the accumulation of cytoplasmic double-stranded DNA, which subsequently triggers hyperactivation of the cGAS-STING interferon signaling pathway, contributing significantly to the pathogenesis of autoimmune disorders (37). Recent evidence (38–40) indicates that TREX1 plays a significant role in cancer progression and immune evasion. For instance, increased TREX1 expression enables pancreatic ductal adenocarcinoma cells to evade immune detection by inhibiting the cGAS-STING pathway (38). Similarly, elevated TREX1 levels have been observed in drug-resistant small-cell lung cancer, potentially facilitating the survival of therapy-resistant tumor cells (39). In alignment with these findings, our data reveal that TREX1 is overexpressed in prostate cancer tissues and exerts pro-tumorigenic effects *in vitro*. Additionally, higher TREX1 expression correlates with an immune microenvironment characterized by diminished effector cell activity and enrichment of immunoregulatory pathways in our analyses. These observations suggest that TREX1 may represent a promising target for immunotherapeutic intervention in prostate cancer. We hypothesize that TREX1 functions as an “innate immune checkpoint” in prostate cancer by attenuating cGAS-STING signaling, thereby facilitating immune evasion and disease progression; nonetheless, this proposition warrants further experimental validation.

The prognostic signature we have constructed includes TREX1, NOX4, and RRM2, with the latter two also being closely associated with cancer progression. NOX4, a member of the NADPH oxidase enzyme family, constitutes a principal source of reactive oxygen species (ROS) that modulate tumor cell proliferation, migration, and invasion. Elevated expression of NOX4 has been demonstrated to promote fibroblast activation and facilitate stromal-epithelial interactions, thereby contributing to the progression of prostate tumors (41). Moreover, increased NOX4 levels are generally correlated with advanced stages of disease (42, 43). Mechanistically, NOX4 activates ROS-dependent PI3K/AKT signaling and promotes M2 macrophage recruitment, shaping an immunosuppressive microenvironment. Under certain stress conditions, NOX4-derived ROS may also increase ferroptosis sensitivity, linking oxidative stress to regulated cell death (44). Lastly, RRM2, the catalytic subunit of ribonucleotide reductase, is critical for DNA synthesis and repair. Aberrant overexpression of RRM2 is frequently observed in various aggressive malignancies, including prostate cancer, where it facilitates cellular proliferation, epithelial-mesenchymal transition (EMT), and is associated with poor clinical outcomes (45–47). Recent studies have further elucidated that RRM2 contributes to resistance against docetaxel chemotherapy by stabilizing ANXA1 and activating the PI3K/AKT signaling pathway, thereby enhancing tumor cell survival under chemotherapeutic stress (48). Collectively, these findings indicate that TREX1, NOX4, and RRM2 may be significant contributors to the progression of prostate cancer; however, their precise roles and potential therapeutic implications warrant further investigation.

This study is subject to several limitations. Firstly, the retrospective nature of the data sourced from TCGA and GEO, coupled with the relatively small size of the validation cohort, may introduce potential biases. Secondly, our analyses were primarily centered on biochemical recurrence; consequently, the capacity of the IFRG signature’s ability to predict metastatic progression or cancer-specific survival was not directly evaluated due to the scarcity of long-term follow-up data. Thirdly, while we provided functional evidence supporting the role of TREX1 in prostate cancer cells, we did not elucidate the molecular mechanisms through which TREX1 knockdown affects proliferation and invasion. We acknowledge this limitation and suggest that future studies examine whether loss of TREX1 activates the cGAS-STING pathway and boosts interferon signaling in PCa cells, as this could explain the immune-related impact of TREX1. If such activation occurs, the combination of TREX1 inhibition with immune checkpoint blockade may represent a promising strategy for prostate cancer immunotherapy. Furthermore, the roles of NOX4 and RRM2 were not experimentally examined in this study. Although some investigations have addressed their involvement in prostate cancer, the underlying mechanisms remain to be elucidated.

## 5 Conclusion

In summary, we have constructed a novel prognostic model that integrates genes associated with ICD and ferroptosis, which effectively stratifies prostate cancer patients according to their risk of disease progression, as validated in an independent external cohort. Notably, our analysis identified TREX1 as a critical determinant of poor clinical outcome; TREX1 is significantly overexpressed in prostate tumor tissues, and its knockdown markedly inhibits prostate cancer cell proliferation, migration, and invasion *in vitro*. These results underscore the potential of TREX1 as a promising target for immunotherapeutic intervention and suggest that modulation of ICD and ferroptosis pathways may improve the precision of immunotherapy strategies in prostate cancer.

## Data Availability

The datasets presented in this study can be found in online repositories. The names of the repository/repositories and accession number(s) can be found in the article/**Supplementary Material**.
